# Uncovering a Medieval Pogrom: Genetic History of a Jewish Community in Catalonia (Spain)

**DOI:** 10.3390/genes17030358

**Published:** 2026-03-23

**Authors:** Laura Pallarés-Viña, Daniel R. Cuesta-Aguirre, M. Rosa Campoy-Caballero, Núria Armentano, Anna Colet, Assumpció Malgosa, Cristina Santos

**Affiliations:** 1 Grup de Recerca en Antropologia Biològica (GREAB), Unitat d’Antropologia Biològica, Departament of Animal Biology, Plant Biology and Ecology, Faculty of Biosciences, Universitat Autònoma de Barcelona, Bellaterra, 08193 Barcelona, Spain; laura.pallares@uab.cat (L.P.-V.); daniel.ruizdelacuesta@uab.cat (D.R.C.-A.); mariarosa.campoy@uab.cat (M.R.C.-C.); assumpcio.malgosa@uab.cat (A.M.); 2 Museu d’Arqueologia de Catalunya, 08038 Barcelona, Spain; nuriaarmentano@gencat.cat; 3 Museu Comarcal de l’Urgell—Tàrrega, 25300 Tàrrega, Spain; info@museutarrega.cat

**Keywords:** ancient DNA (aDNA), population genomics, medieval period, Jewish populations, admixture analysis, Iberian Peninsula, molecular anthropology

## Abstract

**Background/Objectives.** The Black Death pandemic, combined with the antisemitic climate of 14th-century Europe, led to widespread violence against Jewish communities, including numerous pogroms such as the one in 1348 in Tàrrega (Catalonia, Spain). In the Roquetes necropolis of Tàrrega, six communal graves containing at least sixty-nine individuals, with signs of violence, were dated to the mid-14th century. Based on the hypothesis that Iberian medieval Jewish communities preserve genetic similarities to other ancient and modern Jewish communities, our study aims to provide genomic information on medieval Iberian communities, which to date have been unknown. **Methods.** We analyzed DNA from sixteen individuals from the Roquetes necropolis using Twist ancient DNA enrichment capture. Several paleogenomic analyses based on nuclear DNA and uniparental markers were conducted to determine their genetic relatedness and population origin. **Results**. PCA and ADMIXTURE analyses revealed genetic affinities with ancient and modern Jewish populations. Uniparental markers, which exhibited high diversity, aligned with typical patterns within the Jewish community. The qpAdm modeling suggested that the genetic composition of the Roquetes population can be explained by a mixture of Canaan individuals (0.69) and the Iberian non-Jewish non-Islamic medieval population (0.31). No close genetic kinship was detected, but RHO analyses indicated a certain level of background endogamy. **Conclusions**. This is the first study to report genomic data for medieval Iberian Jews. Our findings reveal genomic affinities of the Roquetes individuals with ancient and modern Jewish populations and corroborate the previous attribution of the burials to victims of the 1348 Tàrrega pogrom.

## 1. Introduction

The Jewish people are an ethnoreligious group that traces their origins back to the Late Bronze Age (approximately 1200 BCE) [1] in the Eastern Mediterranean, particularly in the region corresponding to Canaan territory, part of which corresponds to modern-day Israel [1,2,3]. Over the following millennia, successive migratory and exilic movements—both voluntary and forced—gave rise to what is known as the Jewish Diaspora [4]. One of the earliest mass displacements occurred around 720 BCE, with the exile to Mesopotamia after the Neo-Assyrian Empire’s conquest of the northern Kingdom of Israel [5]. Further mass migrations occurred after the destruction of the First Temple by the Neo-Babylonian Empire in 587 BCE [2], and later, after the destruction of the Second Temple by the Roman Empire in 70 CE, when Jewish people started to disperse across the Mediterranean and into Northern Europe [1]. The Diaspora is responsible for the formation of various Jewish communities, two of the most significant being Ashkenazi, corresponding to the Jewish people of Northern Europe, and Sephardic, corresponding to the Jewish people from the Iberian Peninsula [6,7].

Jewish settlement in the Iberian Peninsula is generally dated to the period following the destruction of the Second Temple in 70 CE [1,8]. After the 6th century, with the establishment of Christianity as the main religion, Jewish communities frequently endured social marginalization, legal restrictions, forced conversions, and episodes of expulsion—common phenomena in the European regions where this ethnoreligious group migrated [9,10,11]. During the first period of the Islamic Empire (700–1086 CE), however, the Jewish community experienced a period of relative prosperity, though it —like the Mozarab population (Christians who maintained their religion)—still faced social and institutionalized discrimination. Members of the Jewish community participated in commerce, finance, medicine, and some even partook in governmental structures within the Muslim courts [8,9,12].

This relative stability began to erode with the arrival of the Almoravids in 1086, whose strict orthodoxy curtailed previous tolerance toward *dhimmis* (non-Islamic population protected under a subordinate legal status within Islamic rule), leading to Jewish emigration to northern Christian kingdoms and forced conversions. The situation worsened under the Almohads in the mid-12th century, who imposed severe restrictions. Many Jews converted under duress while secretly maintaining their faith, and others fled north, effectively ending Jewish cultural prosperity in Al-Andalus [8,9]. In contrast, the Jewish communities of northeast Iberia, the Crown of Aragon, developed their own distinctive trajectory. These communities became centers of intellectual and economic activity, shaping the cultural and religious life of medieval Catalonia [13].

The discrimination against the Jewish community severely worsened in the 14th century [14]. The combined effects of the Black Death (1347–1351), recurrent famines, violent conflicts, and economic crisis generated a climate of insecurity that translated to extended antisemitism across Europe [4,9]. Jewish people were scapegoated for the plague and accused of hoarding wealth, leading to pogroms, violent assaults on Jewish quarters, throughout the continent [4,9,12]. In the Iberian Peninsula, the hostilities culminated in 1492, with the implementation of the Edict of Expulsion, which forced all unconverted Jews to leave the Crowns of Castile and Aragon (present-day Spain) [15]. The exiled Jewish population from the Iberian Peninsula largely fled to the Mediterranean basin, settling in regions such as North Africa and the Ottoman Empire (including present-day Turkey), being referred to as Sephardic Jews [7,9].

The expulsion of the Jewish people from the Iberian Peninsula represented the conclusion of this severely antisemitic climate, characterized by numerous episodes of extreme violence. Within the Crown of Castile, the 1391 Seville pogrom spread across the peninsula, resulting in the death of thousands of Jews and mirroring earlier outbreaks of violence, such as those that occurred in the Crown of Aragon in 1348 following the arrival of the Black Death [9]. In the Crown of Aragon (eastern Iberian Peninsula), the most affected cities by the pogroms associated with the pandemic were Barcelona, Cervera, Lleida, Girona, and Tàrrega [9]. Historical sources chronicle a massacre in Tàrrega in 1348, during which armed attackers entered the Jewish quarter and assaulted houses, destroyed deeds, and caused the death of approximately 300 people [16].

**Figure 1:**
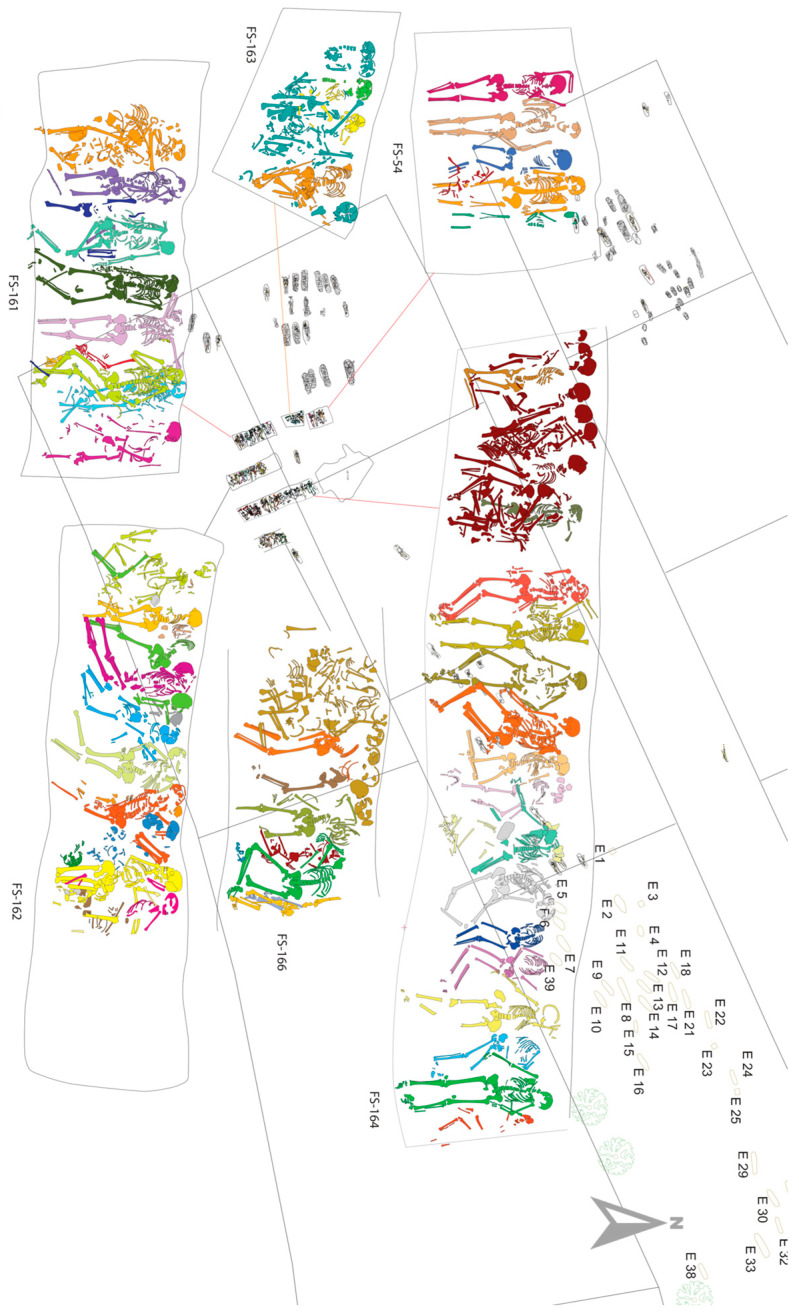
Location of the mass graves of the Roquetes necropolis. For this study, individuals from each mass grave were selected. North is indicated in gray for the general site, not for the close-up of the mass graves. Colors serve as an aid to identify the individualized skeletal elements. Modified from [17].

In 2007, six mass graves dating back to the mid-14th century were found in the Roquetes necropolis (Figure 1) in Tàrrega—part of the Crown of Aragon—suggested by historical data to have belonged to the medieval Jewish community. A total of 69 individuals were recovered from the common graves, although it is suspected that the unexcavated areas of the necropolis hold more mass graves [17]. The typology of the funerary ritual indicated the possibility of a synchronic mass death event. Furthermore, the presence of perimortem traumatisms in at least one third of the individuals, mostly affecting the cranial region, indicated an intentional cause of death, strongly supporting the hypothesis that these burials belonged to victims of the medieval Tàrrega pogrom [18].

Recent advances in paleogenomics provide powerful tools to investigate such historical events and the populations affected by them. Due to centuries of relative social isolation, Jewish communities display genetic continuity between ancient and modern populations [19,20,21,22]. Two recent studies have analyzed genome-wide data from medieval Jewish cemeteries: one from Norwich, England [21]—where individuals were victims of an antisemitic massacre—and another from Erfurt, Germany [20]. Both studies revealed populations with mixed Middle Eastern and European ancestry, as well as genetic continuity between the studied medieval Jewish individuals and present-day Ashkenazi Jews.

This study aims to analyze the genetic profiles of individuals recovered in the necropolis of Roquetes. By applying advanced paleogenomic tools, we seek to address the current absence of genomic data on medieval Iberian Jewish populations and to provide the first genomic information for these communities, which remain completely unknown from a genomic perspective.

## 2. Materials and Methods

### 2.1. Sample Preparation, DNA Extraction, and Quantification

Bones from sixteen individuals (Table 1), from different osteological sex and age groups, exhumed from all six mass graves at the Roquetes necropolis (Tàrrega, Catalonia, Spain) were selected (Supplementary Material, Table S1). The outer surface of each bone was initially cleaned to remove dirt and then polished with a milling cutter to eliminate the outermost layers. A 1 cm section was selected and pulverized using a hammer for subsequent DNA extraction.

For the petrous bones, 3D photogrammetry models were created prior to pulverization, ensuring the creation of precise digital replicas that capture their detailed morphology.

Around 100–150 mg of bone powder was used for DNA extraction in a 48 h incubation with a solution of Proteinase-K and EDTA, and eluted in 60 µL of EBT as in Vinueza-Espinosa et al. (2020) [23]. Extracted DNA was purified as described by Vinueza-Espinosa et al. (2020) [23], using the High Pure Viral Nucleic Acid Large Volume Kit (Roche), a silica in HE-membrane column-based method. Blank controls were processed with each extraction. A quantitative PCR (qPCR) of a small mitochondrial DNA fragment was performed to confirm the absence of contamination during DNA extraction. Qubit^®^ dsDNA HS Assay Kit (ThermoFisher Scientific, Waltham, MA, USA) was used to quantify each sample, and Bioanalyzer^®^ Agilent High Sensitivity DNA Kit (Agilent Technologies, Barcelona, Spain) was employed to obtain fragmentation profiles.

All sample preparation and processing steps were conducted in the Biological Anthropology Unit’s dedicated clean ancient DNA laboratory at the Universitat Autònoma de Barcelona (UAB).

### 2.2. Library Preparation, Sequencing, and Enrichment

Twenty µL of extracted DNA was used to perform single-strand libraries with the Santa Cruz Reaction (SCR) protocol [24]. For each sample, 50 µL of the library was recovered and purified using the MinElute PCR Purification Kit (Qiagen, Barcelona, Spain). The indexing PCR cycle number was estimated by qPCR using QuantiNova^TM^ SYBR Green PCR Kit (Qiagen). Overall, 22.5 µL of the library was indexed using the KAPA HiFi HotStart Uracil+ kit (Roche) with the following program: initial denaturalization 3′ at 95 °C; X cycles 20″ at 98 °C, 30″ at 60 °C, 30″ at 72 °C; final incubation 7′ at 72 °C, 4 °C forever. Indexed P7 and indexed P5 primers for the Illumina platform, similar to Kircher et al. (2012 [25]) were used to perform unique dual-index sequencing.

Finally, the amplified libraries were purified using SPRIselect beads (Beckman Coulter, Brea, CA, USA) and quantified with the Qubit^®^ dsDNA HS Assay Kit (ThermoFisher Scientific). Bioanalyzer^®^ Agilent High Sensitivity DNA Kit (Agilent Technologies) was employed to obtain the library size profile. The amplified and indexed libraries were then pooled in equimolar amounts and sequenced with Illumina technology.

Libraries were enriched with the Ancient Human DNA Target Enrichment kit (Twist Biosciences, San Francisco, CA, USA), which includes DNA baits targeting the same ~1.3 million nuclear SNPs from the 1240k panel [26], following the manufacturer’s protocol. After, libraries were pooled in equimolar amounts and sequenced with Illumina technology in a NextSeq 1000 sequencer (Illumina, San Diego, CA, USA).

### 2.3. aDNA Data Processing and Authentication of Whole Genome Sequencing

After demultiplexing based on a dual-unique index combination developed by the sequence provider, raw sequencing data were processed with the nf-core/eager pipeline v2.4.7 [27,28]. Adapters were removed with AdapterRemoval v2.3.2 [29], and sequences shorter than 30 bp were eliminated. Then, sequences were mapped against the Human Reference Genome GRCh37 (hg19, GenBank accession GCA_000001405.1) with BWA v0.7.17-r1188v [30], aln, and samse/sampe commands. Parameters “-l 1024-n 0.01-o 2” were used, following recommendations from Oliva and colleagues [31,32]. SAMtools v1.12 [33] was used to generate sorted BAM files with all the reads with a mapping quality of over 20. Duplicates were removed with the MarkDuplicates tool from Picard v2.26.0 software [34]. Damage levels were assessed with PMDTtools v0.50 [35]. Finally, seven bases were trimmed at the start/end of the reads with BamUtils v1.1.2 [36] to avoid ancient damage in final genotypes. The quality of FASTQ files was evaluated with FastQC v0.11.9 [37], and the quality of the BAM file was analyzed with QualiMap v2.2.2-dev [37].

### 2.4. Sex Determination, Contamination, and Genotyping

Genetic sex was determined based on the relative coverage of specific X and Y chromosomes’ SNPs using the tool Sex.DetERRmine.py v1.1.2 [38], implemented in the nf-core/eager pipeline [27,28].

Ancient DNA authenticity was evaluated for all samples using several criteria [39]: A rate of cytosine deamination at the terminal nucleotide above ~10%, since non Uracil-DNA glycosylase treatment was used; a ratio of Y to combined X + Y chromosome sequences below 0.03 or above 0.35, where intermediate values are indicative of the presence of DNA from at least two individuals of different sex; and an upper bound rate for the 95% confidence interval for the rate to the consensus mitochondrial sequence that exceeds 95%, as computed using contamMix-1.0.10 [40]. Additionally, mtDNA was manually reviewed for inconsistencies. We directly discard samples with a rate of cytosine deamination at the terminal nucleotide lower than 5%, or samples with at least two signals of contamination.

Trimmed BAM files were used to call genotypes with pileupCaller, which generates pseudo-haploid genotypes by randomly choosing one allele at every SNP position. The panel with 1240k nuclear SNPs used in ancient population genomics was used [26].

### 2.5. Mitochondrial and Y Chromosome Haplogroups Assignment

Mitochondrial reads that had already been mapped with the mitochondrial DNA for the hg19 genome were realigned against the revised Cambridge Reference Sequence (rCRS, GenBank accession NC_012920.1) [41] with nf-core/eager pipeline v2.4.6 [27,28]. CircularMapper v1.0 [42] was used to avoid the problems associated with the circularity of the mtDNA molecule. Genotypes were called with freebayes v1.3.6 [43] and vcflib v1.0.3 [44]. Haplogrep 3 v3.2.1 [45] was used to call mitochondrial DNA haplogroups. SNPs were manually double-checked, visualizing alignments with the Integrative Genome Viewer (IGV) v2.11.2 [46]. Results were considered reliable at a coverage of 3X after visual confirmation. In the low-coverage regions, the damage, mutational, and heteroplasmic profiles [47,48,49,50] were examined to improve credibility.

For males, Yleaf v3.2.1 [51] with parameters-r 2 (minimum number of reads for each base), -q 30 (minimum quality for each read), -b 60 (minimum of a base result for acceptance), -dh (draw the haplogroups in the haplogroup tree), and -p (search for private mutations), was used to determine the Y-chromosome haplogroup. The trimmed BAM files were used.

### 2.6. Genetic Relatedness

Autosomal SNPs were used to define first- and second-degree relatedness among pairs of individuals based on their mismatch rate (PMR), using the tool BREADR v1.0.2 [52]. Specifically, the pairs of individuals were categorized as “Same/Twins”, “First-degree”, “Second-degree”, or “Unrelated” (in the first or second degree), and only pairs with at least 500 shared SNPs were trusted.

### 2.7. Principal Components Analysis (PCA)

Newly reported samples were merged with previously published datasets of ancient and modern individuals from the Allen Ancient DNA Resource (AADR) [53]. Only one of the individuals was considered when there was a first- or second-degree of relatedness. Samples from Human Origins [54] were also included for principal component analysis (PCA). Ancient European Jewish samples from Norwich, UK [21] and Erfurt, Germany [20] were compiled and added. Samples were labeled for each analysis as indicated in the Supplementary Material (Table S2).

The PCA was performed using the smartpca software from EIGENSOFT v8.0.096,97 with the “lsqproject” and “shrinkmode” options activated (YES) and a list of modern populations from Europe, North Africa, Caucasus, and Southwestern Asia (Supplementary Material, Table S2). All ancient individuals, including those from the medieval period of the Iberian Peninsula, were projected onto PC1 and PC2.

### 2.8. f-Statistics

The *f*-statistics were performed with ADMIXTOOLS v5.1 (https://github.com/DReichLab/AdmixTools, accessed on 28 October 2025) and plotted with R v4.4.2 [55] using the ggplot2 v3.5.1 package [56].

Outgroup *f*_3_-statistics, computed using qp3Pop with the ‘inbreed’ parameter activated, measured the shared genetic drift between two populations (A and B) relative to an outgroup (C), providing a similarity measure: higher values indicate greater genetic similarity between A and B. In this study, we calculated outgroup *f*_3_-statistics in the form *f*_3_ (Roquetes, Test; Mbuti), where the test populations were present-day and ancient Jewish populations and non-Jewish Medieval populations from the Iberian Peninsula, with Mbuti, a present-day Sub-Saharan hunter-gatherer population from the Democratic Republic of Congo, being used as the outgroup.

Moreover, for each present and ancient Jewish population, and for the non-Jewish Medieval populations from the Iberian Peninsula, their genetic similarity was quantified using the outgroup *f*_3_-statistic of the form *f*_3_ (Pop1, Pop2; Mbuti), calculated with qp3Pop from ADMIXTOOLS v5.1 (https://github.com/DReichLab/AdmixTools, accessed on 28 October 2025). The *f*_3_ results were converted into a pairwise distance matrix where each cell represented 1-*f*_3_ (Pop1, Pop2; Mbuti), such that smaller values corresponded to higher shared genetic drift. The resulting matrix was symmetric, and diagonal values were set to zero to preserve matrix completeness. Multidimensional scaling (MDS) was then performed using the classical metric scaling function “cdmscale” in R v4.4.2 [55] (base stats package), with k = 2 dimensions and otherwise default parameters. The eigenvalues returned by “cdmscale” were used to calculate the proportion of variance explained by each of the first two MDS dimensions. The MDS coordinates were visualized using ggplot2 v3.5.1 [56].

### 2.9. Admixture Modeling

Related individuals were excluded from admixture modeling analyses. ADMIXTURE v1.3.0 was used to define the main genetic cluster profiles [54]. Five populations, presenting differential relatedness between them and the Roquetes population, were selected as fixed reference groups of ancestry (k = 5) to implement a supervised ADMIXTURE model [54]:

Western Hunter-Gatherers: a group of Mesolithic hunter-gatherers who scattered over western, southern, and central Europe.

Iberomaurusian: a Late Pleistocene population from Morocco, accounts for potential North African-related ancestry to improve differentiation between southern European, Levantine, and North African genetic components.

Anatolia_Neolithic: population of farmers that scattered from Anatolia to West Eurasia around 7000BC.

Russia_Samara_Early Bronze Age_Yamnaya: a population, associated with the Yamnaya culture, originated in the Samara Bend of present-day Russia, dating from 5000 to 4000 BP. They present both an Eastern hunter-gatherer component and a Caucasus hunter-gatherer component.

Israel_Middle Late Bronze Age: population from Canaan, territory of present-day Israel, dating from the Middle Late Bronze Age (MLBA), the period of time associated with the emergence of Judaism.

The model was replicated twenty times, and the results were merged with Pong v1.5 [57].

We modeled ancestry using qpAdm from the ADMIXTOOLS v5.1 package (https://github.com/DReichLab, accessed on 5 November 2025), enabling “allsnps: YES” to maximize the number of SNPs used (and thus the power to reject implausible models). We follow the terminology and framework of Harney et al. (2021) [58] in which qpAdm evaluates whether a target population can be represented as a mixture of one or more Source populations, using a panel of reference populations to differentiate shared genetic drift among the source population groups.

In qpAdm, the reference populations serve as statistical outgroups: they are selected to be differentially related to the source populations to provide leverage to separate their ancestry contributions. Although they are not phylogenetic outgroups in the strict sense, they function as outgroups within the qpAdm framework by anchoring deep ancestry components and breaking symmetries among the source groups [58].

Following Harney et al. (2021) [58], we interpret qpAdm *p*-values as model diagnostics: models with *p* > 0.05 are termed plausible, whereas models with *p* ≤ 0.05 are implausible and are therefore rejected. We describe models as plausible/rejected under the qpAdm framework.

We first evaluated one-way models to test whether the target could be adequately modeled as deriving entirely from a single source. Conceptually, a plausible one-way model indicates that the target is indistinguishable from forming a clade with that source relative to the reference panel; it does not imply admixture, but rather identity or near identity of ancestry profiles in the qpAdm sense. We then tested two-way models to evaluate whether the Roquetes population could be explained as a mixture of Canaan (labeled as Israel MLBA) individuals and non-Jewish medieval Iberian populations.

For all the models applied here, we used a set of 4 outgroups (reference populations) [58]:

Mbuti, a present-day Sub-Saharan hunter-gatherer population from the Democratic Republic of Congo, serves as a deep non-West Eurasian outgroup that helps distinguish West Eurasian ancestry from older African lineages without introducing the long-branch biases associated with chimpanzee outgroups.

Ust’-Ishim, an Upper Paleolithic individual from western Siberia (~45 ka), represents an early non-African lineage and provides resolution among West Eurasian populations before their subsequent diversifications.

Iberomaurusian, a population previously presented.

Villabruna, a Mesolithic hunter-gatherer sample from northern Italy, represents the Western European hunter-gatherer (WHG) ancestry component and provides sensitivity to differential relatedness among European-derived populations.

### 2.10. Runs of Homozygosity (ROH)

To assess demographic and inbreeding patterns in the population, we examined runs of homozygosity (ROH), defined as stretches of the genome that lack genetic variation and thus reflect the coinheritance of identical haplotypes from related parents. Software hapROH v0.64 [59] was used with default settings to identify runs of homozygosity within the genome of individuals with more than 400,000 autosomal SNPs covered from the 1240k panel [26]. Following Ringbauer et al.’s (2021) [59] indications, ROH were categorized in the following longitude intervals (in centimorgans-cM): 4–8 cM, 8–12 cM, 12–20 cM, and 20–300 cM.

## 3. Results

### 3.1. Quality of the Samples

Overall, it was possible to sequence samples of most of the individuals, and only one sample presented signs of being contaminated. Twelve out of the sixteen individuals available presented enough short fragments (50–220 bp) in the extracted DNA for generating single-strand libraries (Supplementary Material, Table S1). The library corresponding to ROQ 8 was discarded for being considered contaminated, as it presented 4.30% of C-to-T changes at the terminal nucleotide (Supplementary Material, Table S3). The remaining eleven libraries presented no signs of contamination: a rate of cytosine deamination at the terminal nucleotide above ~10%, indicative of post-mortem damage expected in ancient DNA; a ratio of Y to combined X + Y chromosome sequences consistent with the presence of one single individual; and a confidence rate for the contamMix-1.0.10 [40] (an upper bound rate for the 95% confidence interval for the rate to the consensus mitochondrial sequence that exceeds 95%) (Supplementary Material, Table S3). Most of these samples (ROQ1, ROQ5, ROQ7, ROQ10, ROQ11, ROQ12, and ROQ13) presented levels of endogenous DNA below 0.5%. Three samples (ROQ2, ROQ3, and ROQ4) presented endogenous DNA percentages between 5 and 20% (Supplementary Material, Table S3), and one (ROQ15) had a percentage of endogenous DNA of 63.77% (Supplementary Material, Table S3), yielding a considerable number of SNPs (121,312 SNPs) from the 1240k panel [26].

For the ten samples with a percentage of endogenous DNA lower than 20% (Supplementary Material, Table S3), DNA capture procedures were applied, substantially increasing the proportion of endogenous DNA (Supplementary Material, Table S4). A minimum of approximately 50,000 SNPs was recovered per captured sample, with several achieving much higher values—for instance, ROQ2 and ROQ4 yielded over 990,000 and 1,000,000 SNPs, respectively (Supplementary Material, Table S4).

### 3.2. Genetic Sex, Genetic Relatedness, and Endogamy

The genetic sex was estimated for the eleven individuals, obtaining four males and seven females. BREADR genetic relatedness analyses revealed no first- or second-degree relationships between the analyzed individuals from Roquetes (Supplementary Material, Table S5).

**Figure 2:**
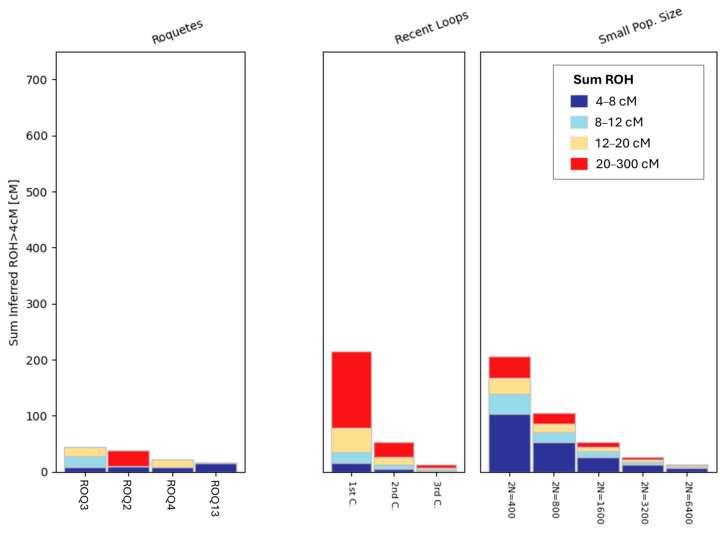
Runs of Homozygosity (ROH) analysis exhibiting cumulative ROH lengths exceeding 4 cM for individuals with more than 400.000 autosomal SNPs.

Results of the analysis of runs of homozygosity (ROH) revealed the presence of some medium and long homozygous regions (Figure 2 and Figures S1–S4), and put the focus on individual ROQ2, which exhibited long ROHs (20–300 cM), indicating background endogamy.

### 3.3. Uniparental Markers

High mitochondrial diversity was identified, with ten different haplogroups in twelve unrelated individuals (Table 2 and Supplementary Material, Table S6). The two repeated haplogroups (L2a1c + 16129 and H1bo) belonged to individuals without evidence of close genetic relatedness (Supplementary Material, Table S5), despite sharing their mitochondrial haplotypes (Supplementary Material, Table S6).

The mtDNA haplogroups identified in the Roquetes individuals (Table 2) were contextualized by assessing their presence in ancient populations using the Allen Ancient DNA Resource (AADR v62.0) [53] and by evaluating their occurrence in present-day Jewish populations based on published data [60]. Several haplogroups detected in Roquetes have been previously reported in ancient Jewish contexts, including K1a1b1a and U5a2b, both identified among medieval Jews from Erfurt, as well as U5a2b2a in modern Ashkenazi populations (Table 2). Other haplogroups, such as M1a1b1c, J1c1, R0a4, and H1bo, have not been directly observed in the AADR database but are present in modern Jewish populations, particularly among Ashkenazi and Sephardic groups (Table 2). In some cases, closely related parent haplogroups (e.g., M1a1b1 or H20a) have been identified in ancient individuals from the Iberian Peninsula, the eastern Mediterranean, or the Near East, suggesting broader Mediterranean or Near Eastern maternal lineages (Table 2).

The Y-chromosome haplogroups identified in the male individuals from Roquetes (Table 3 and Supplementary Material, Table S10 and Figures S5–S8) were contextualized using the Allen Ancient DNA Resource (AADR v62.0) [53]. The detected lineages (J, E, and G) show diverse geographic and temporal distributions in the ancient record. Haplogroup J2a2a (ROQ1) and E1b1b (ROQ2) have broad distributions in the Mediterranean and Near East, with E1b1b also reported in medieval Jewish populations from Norwich and Erfurt (Table 3). The G1a1a lineage identified in ROQ4 has been documented in Chalcolithic and Bronze Age Iran and in historical-period Turkey, suggesting an eastern origin (Table 3). Haplogroup E2a (ROQ7) is rare in ancient DNA datasets and has been reported in a limited number of ancient and present-day individuals from eastern Africa (Table 3).

### 3.4. Exploratory Data Analysis: PCA and ADMIXTURE

**Figure 3:**
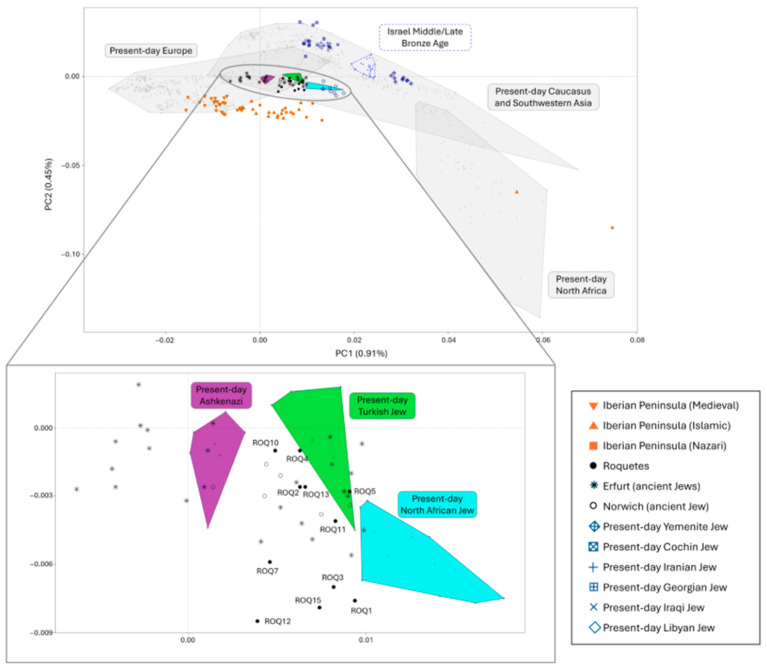
Principal Component Analysis (PCA) of present-day European, Caucasus, Southwestern Asian, and North African populations, with ancient individuals projected onto PC1 and PC2. Canaan individuals (labeled as Israel Middle/Late Bronze Age) are outlined by a blue dashed polygon. Filled polygons represent present-day Jewish populations from Turkey (Sephardic), North Africa, and Ashkenazi Jews. Other present-day Jewish populations are shown as blue dots, with point shape indicating geographic origin (see legend). Ancient Jewish individuals are shown in black: an open circle for Norwich, an asterisk for Erfurt, and a filled circle for Roquetes. Non-Jewish medieval individuals from the Iberian Peninsula are shown in orange, with the shape indicating their culture.

To investigate the genetic profile of the Roquetes population, we merged our dataset with a collection of present-day populations and medieval Jewish individuals from Erfurt and Norwich (see Supplementary Material, Table S2 for labels). These individuals were projected onto the first principal components (PC1 and PC2) of a Principal Component Analysis (PCA) based on present-day populations from Europe, North Africa, the Caucasus, and Southwestern Asia (Figure 3). After filtering, 553811 autosomal SNPs from the 1240k panel [26] remained for analysis. The Roquetes individuals overlapped with present-day Turkish Jews (Sephardic), as well as with the ancient Jewish individuals from Erfurt and Norwich (Figure 3). Conversely, Roquetes individuals were genetically distant from the non-Jewish Iberian medieval population (Figure 3).

**Figure 4:**
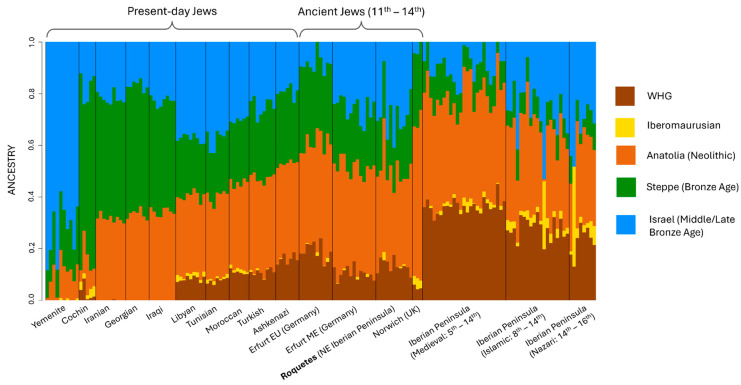
Ancestry proportions using ADMIXTURE modeling for different populations, the Roquetes population highlighted in bold. The labels *Erfurt (EU)* and *Erfurt (ME)* refer to medieval Jewish individuals from Erfurt of European and Middle Eastern descent, respectively, and the label Israel Middle/Late Bronze Age refers to individuals from Canaan (territory of present-day Israel in the MLBA).

ADMIXTURE analyses suggested the Roquetes population’s ancestry could be modeled as a mixture of Western hunter-gatherer (WHG), Steppe Bronze Age, and Anatolian Neolithic components, together with ancestry related to populations from Canaan (territory of present-day Israel in the MLBA), and a minor Iberomaurusian contribution for some individuals (Figure 4). This ancestral profile closely resembles that observed in the Jewish individuals from medieval Erfurt.

### 3.5. Formal Tests of Genetic Affinity and Admixture

**Figure 5:**
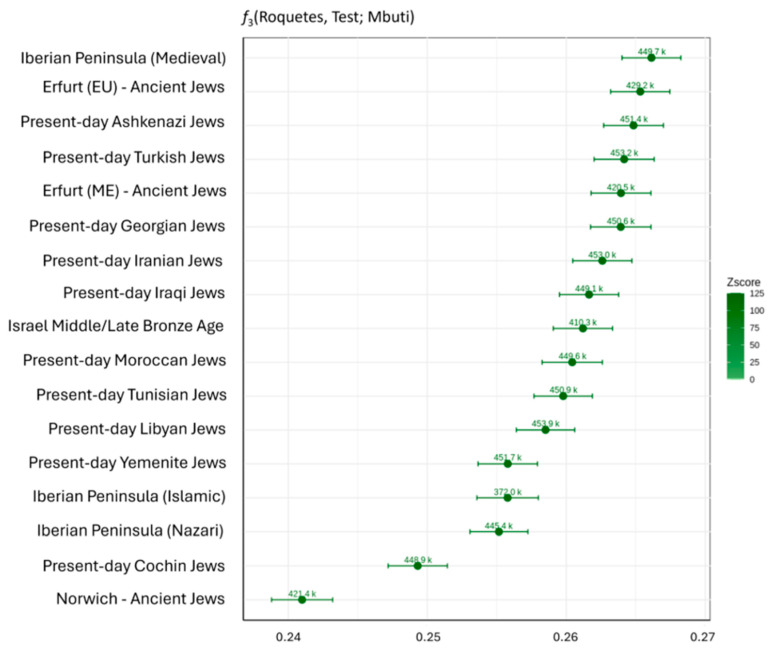
Outgroup *f*_3_-statistics in the form *f*_3_(Roquetes, Test; Mbuti) for the Roquetes site. Color represents the Z-score values, with a minimum Z-score of 109.455. The number of SNPs is displayed in a shortened format, where values are expressed in thousands. The labels *Erfurt (EU)* and *Erfurt (ME)* refer to medieval Jewish individuals from Erfurt of European and Middle Eastern descent, respectively, and the label Israel Middle/Late Bronze Age refers to individuals from Canaan (territory of present-day Israel in the MLBA). Results showed that Roquetes individuals presented the highest shared genetic drift with non-Jewish non-Islamic Medieval individuals from the Iberian Peninsula, ancient Ashkenazi Jews from Erfurt (Germany), and present-day Ashkenazi and Sephardic (Turkey) Jews. All results are displayed in Supplementary Material (Table S13).

In order to identify the closest genetic matches for the medieval Jews from the Roquetes and further assess the PCA and ADMIXTURE-based observations, we first performed outgroup *f*_3_-statistics in the form *f*_3_ (Roquetes, Test; Mbuti) using individuals with more than 50,000 autosomal SNPs (Figure 5 and Supplementary Material, Table S13). Our results indicated that Roquetes individuals shared higher genetic drift with non-Jewish non-Islamic Medieval population from the Iberian Peninsula and Medieval Ashkenazi Jews from Erfurt (Germany), as well as with present-day Ashkenazi and Sephardic (Turkey) Jewish populations (Figure 5 and Supplementary Material, Table S13). In contrast, lower genetic affinity was observed with the Islamic and the Nazari (the last Islamic dynasty of Al-Andalus [9]) individuals from the Iberian Peninsula and Medieval Norwich (UK) Jews, as well as with other present-day Asian Jews, such as Cochin (India) or Yemenite (Yemen) Jews (Figure 5 and Supplementary Material, Table S13).

**Figure 6:**
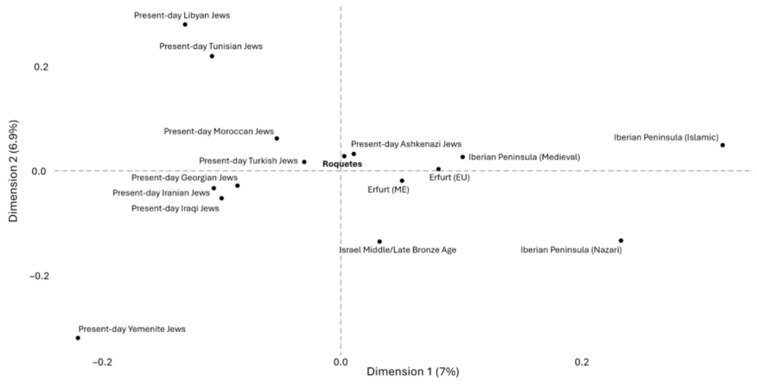
Multidimensional scaling (MDS) based on the 1—outgroup *f*_3_ in the form *f*_3_(Pop1, Pop2; Mbuti) for the populations employed in the present study and removing the outlier populations, namely present-day Cochin Jews and ancient Norwich (UK) Jews (The MDS including all populations is displayed in Supplementary Material, Figure S9). The labels *Erfurt (EU)* and *Erfurt (ME)* refer to medieval Jewish individuals from Erfurt of European and Middle Eastern descent, respectively, and the label Israel Middle/Late Bronze Age refers to individuals from Canaan (territory of present-day Israel in the MLBA). All results are displayed in Supplementary Material (Table S14).

The MDS based on pairwise 1–*f*_3_ distances largely confirms the affinities observed in the PCA and outgroup *f*_3_ analyses (Figure 6 and Supplementary Material, Table S14 and Figure S9). Roquetes individuals clustered closely with present-day Ashkenazi Jews and Sephardic Jews from Turkey, as well as with the medieval Ashkenazi individuals from Erfurt (Germany), indicating elevated levels of shared genetic drift among these groups. Non-Jewish non-Islamic Medieval population from the Iberian Peninsula occupied a nearby position in the MDS space, consistent with the high *f*_3_ values observed in pairwise comparisons.

To formally test whether the genetic affinities inferred from PCA, ADMIXTURE, outgroup *f*_3_-statistics, and MDS could be explained by specific ancestry models, we performed qpAdm analyses using one- and two-way admixture scenarios, always using the same group of outgroups (reference populations). For the one-way models, we tested whether the Roquetes population could be plausibly modeled from a single source population, including non-Jewish non-Islamic medieval individuals from the Iberian Peninsula, Islamic and Nazari medieval Iberian populations, medieval Jewish individuals from Erfurt —separated into individuals of European (EU) and Middle Eastern (ME) genetic origin—, medieval Jewish individuals from Norwich, Canaan (territory of present-day Israel in the MLBA) Age individuals, and present-day Jewish populations from Turkey (Sephardic), Ashkenazi, and Morocco (Supplementary Material, Table S15). Among these models, only the Erfurt medieval Jewish population with Middle Eastern genetic affinity yielded a plausible fit (*p* = 0.098), whereas the other one-way models were rejected since the *p*-value was lower than 0.05 (Supplementary Material, Table S15).

**Figure 7:**
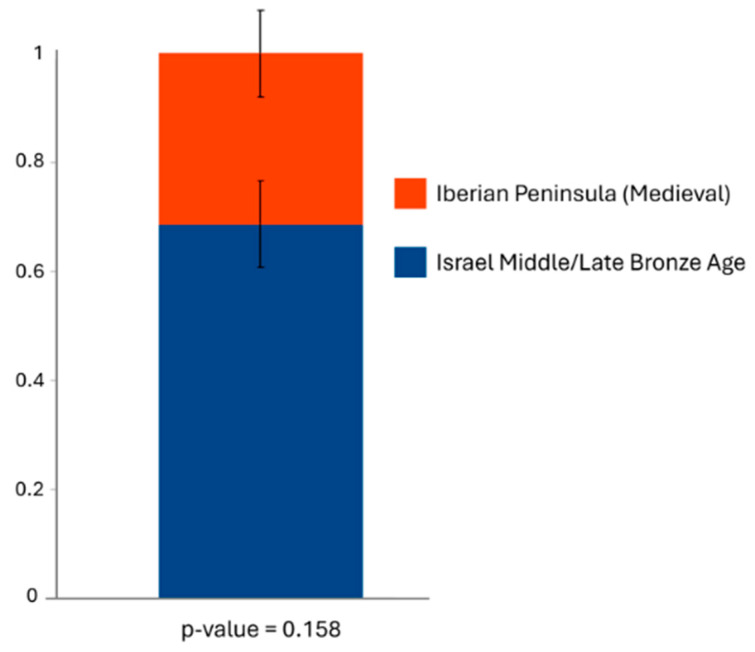
Plausible two-way qpAdm model for the population of Roquetes (n = 12). The *p*-value is indicated under the model. The raw data is displayed in the Supplementary Material (Table S15). Canaan individuals are labeled as the Israel Middle/Late Bronze Age. The raw data is displayed in the Supplementary Material (Table S15).

We tested whether the Roquetes populations could be modeled as a mixture of individuals from Canaan (labeled as Israel Middle/Late Bronze Age) and medieval populations from the Iberian Peninsula, including non-Jewish non-Islamic, Islamic, and Nazari groups, using two-way models (Supplementary Material, Table S15). Among the tested models, only the combination of Canaan individuals (labeled as Israel Middle/Late Bronze Age) and the non-Jewish non-Islamic medieval population from the Iberian Peninsula provided a plausible fit (*p*-value = 0.158), with estimated ancestry proportions of 0.686 and 0.314, respectively (Figure 7 and Supplementary Material, Table S15).

These results indicate strong genetic affinity between the Roquetes population and the Erfurt medieval Jewish population with Middle Eastern genetic affinity, and provide a plausible model of ancestry by combining Canaan individuals and the non-Jewish non-Islamic medieval population from the Iberian Peninsula.

## 4. Discussion

We analyzed genome-wide and uniparental data from 16 individuals exhumed from the Roquetes necropolis (Tàrrega, Catalonia, Spain), including the six mass graves identified previously, securely dated to the mid-14th century, and archeologically interpreted as a single catastrophic episode [17,18]. While the sample covers only a subset of the excavated individuals, multiple lines of evidence converge on a non-familial burial event: we find no first- or second-degree genetic kinship among analyzed individuals, and a significant fraction of the broader burial assemblage exhibits perimortem cranial trauma [18]. Genomic data cannot identify the cause of death; however, it revealed affinity with ancient and present-day Jewish communities that, when integrated with the archeological context, dating, and historical sources for 1348, reinforce the attribution to the Tàrrega pogrom.

Following ancient Jewish law, religious affiliation is maternally assigned [70]; consequently, mtDNA variation can offer complementary insights into Jewish community histories and origins. The Roquetes individuals show high mitochondrial diversity (haplogroups H, J, K, L, M, R, U), contrasting with the narrow mtDNA spectra documented in medieval Ashkenazi communities at Erfurt (Germany), where strong founder effects and drift operated [20], and at Norwich, where elevated levels of consanguinity could also have influenced the genetic drift [21]. The pattern observed in Roquetes aligns more closely with Sephardic-descended communities [71,72], where modern Jewish populations that received exiles from Iberia—Bulgaria, Turkey, and Morocco—display substantial haplogroup diversity, in contrast to other Jewish groups (e.g., Azerbaijan, Georgia, Libya, Mumbai (India), and Belmonte (Portugal)) that exhibit bottleneck signatures [19,71,73,74].

Notably, we observe haplogroups with strong Jewish associations or prior medieval occurrences—e.g., K1a1b1a (common in Ashkenazi; present in Erfurt), U5a2b, and M1a1b1c (present in Erfurt), with broader presences of H1 lineages, R0a, J1 subclades in Jewish and non-Jewish populations [20,21,60,75]. Their occurrence in Roquetes does not imply exclusivity to Jewish groups but, in combination with autosomal affinity patterns, highlights that Iberian Jewish communities maintained higher mtDNA heterogeneity than contemporaneous Central/Northern European groups [19,74,76].

Among the four males, we detect J2 (J2a2a1~*), E (E-CTS9507; E-Y231455), and G (G-PH1944/FT19393) haplogroups. These lineages have deep Neolithic–Bronze Age roots in the Eastern Mediterranean/Levant and adjacent regions and are frequent in Jewish diasporic contexts [19,66,77,78]. The recurrence of E1b1b-lineages in medieval Jewish cemeteries (Erfurt, Norwich) and the presence of J and G among present-day Jewish groups further underscore continuity from Levantine–Mediterranean ancestry sources [20,21,78,79,80]. Given the constraints of coverage and marker density in ancient Y-chromosome inference, subclade resolution should be interpreted cautiously; nonetheless, the observed diversity argues against a tight, highly drifted paternal pool at Roquetes.

PCA places Roquetes in proximity to medieval Jewish samples from Erfurt/Norwich and to modern Sephardic Jews (e.g., Turkey, North Africa), while distinct from non-Jewish medieval Iberians. Supervised ADMIXTURE (k = 5) highlights a combination of Anatolian Neolithic, Steppe Bronze Age, and Canaan (territory of present-day Israel in the MLBA)-related components, with minor Iberomaurusian signals in some individuals, an ancestry palette recurrent in Jewish diaspora datasets [20,22]. Importantly, ADMIXTURE components are not population-specific and can reflect shared deep ancestries across the Mediterranean (e.g., Levantine-related contributions also present in North Africa via Phoenician and later historical contacts). We therefore rely on formal tests for specificity. In this line of evidence, outgroup *f*_3_ statistics in the form of *f*_3_ (Roquetes, Test; Mbuti) and MDS on 1–*f*_3_ distances confirm elevated shared drift with medieval Erfurt Jews and with modern Jewish groups (Ashkenazi; Sephardic from Turkey), and comparatively reduced affinity to Islamic/Nazarí medieval Iberians and to Asian Jewish groups (e.g., Cochin, Yemenite) [20,21]. The apparent genetic distance to Norwich is compatible with its strong drift/consanguinity, which can accentuate separation in low-dimensional summaries [21].

Crucially, qpAdm identifies two-way models that fit the Roquetes population as a mixture of Canaan (labeled as Israel Middle/Late Bronze Age) and non-Jewish non-Islamic medieval Iberian populations (*p* = 0.158), with point estimates around ~0.69 for Levantine ancestry and ~0.31 for Iberian medieval ancestry. Among one-way models, only the Erfurt subgroup with Middle Eastern affinity is marginally plausible (*p* = 0.098), consistent with a Levantine-centered core plus Iberian admixture. Together, these results indicate that the Roquetes community preserved a distinctively Jewish genetic signature while incorporating local Iberian ancestry.

Genetic evidence for limited but not zero admixture at Roquetes correlates with historical records: conversions and interfaith relationships occurred across medieval Iberia despite religious proscriptions [81,82,83]. On broader scales, present-day Jewish groups—including Sephardic—harbor substantial European admixture (~30–60%) [22,84,85,86,87,88], and Sephardic ancestry persists detectably in modern Iberians [79,89]. The mtDNA diversity we document aligns with the Sephardic pattern [19] and contrasts with communities shaped by founder effects [19,21].

As mentioned before, kinship analyses show no first- or second-degree relationships, indicating that the mass burials do not reflect family-based interments. Moreover, ROH profiles reveal background endogamy in one case (ROQ2), which exhibits long ROH segments that could be compatible with mating patterns in relatively small or socially insular communities [59]. This mirrors patterns seen in other medieval Jewish contexts yet remains milder than the strong consanguinity reported in Norwich [21].

## 5. Conclusions

This is the first study ever to report genomic data for medieval Iberian Jews. Across uniparental and autosomal lines, the Roquetes individuals exhibit (i) Eastern Mediterranean/Levantine ancestry consistent with Jewish origins, (ii) mtDNA diversity characteristic of Jewish communities, (iii) paternal lineages common in Jewish diasporas (J2, e.g., (iv) formal models requiring Canaan plus Iberian medieval sources, and (v) endogamy without close kinship. When integrated with the 14th-century context and osteological evidence of lethal violence, these data support the previous attribution of the mass graves to victims of the 1348 Tàrrega pogrom. Beyond the event attribution, the study broadens the genetic portrait of medieval Iberian Jewish communities, underscoring their heterogeneity and regional interactions within Iberia.

## Figures and Tables

**Table 1:** Genetic and archeological code for each individual studied from the necropolis of Roquetes and the corresponding grave.

Genetic Code	Archeological Code	Grave
ROQ 1	FS 164 UE 1210	Mass grave 164
ROQ 2	ROQ′07 FS166 UE 1206 x	Mass grave 166
ROQ 3	FS164 UE 1204	Mass grave 164
ROQ 4	FS164 UE 1220 (7)	Mass grave 164
ROQ 5	ROQ′07 FS164 UE 1222	Mass grave 164
ROQ 6	FS162 UE 1175	Mass grave 162
ROQ 7	ROQ′07 FS164 UE 1218	Mass grave 164
ROQ 8	ROQ′07 FS164 UE 1219	Mass grave 164
ROQ 9	FS162 UE 1167	Mass grave 162
ROQ 10	FS162 UE 1165	Mass grave 162
ROQ 11	FS163 UE 1185	Mass grave 163
ROQ 12	ROQ′07 FS164 UE 1213	Mass grave 164
ROQ 13	FS54 UE 1053 (1)	Mass grave 54
ROQ 14	ROQ′07 FS161 UE 1187	Mass grave 161
ROQ 15	ROQ′23 UF2 UE1009	Individual grave
ROQ 16	ROQ′23 UF1 UE1006	Individual grave

**Table 2:** mtDNA haplogroups from the samples of Roquetes. Their ancient distribution, based on the Allen Ancient DNA Resource (AADR) [53] v62.0, and the present-day distribution among Jewish people [60] are displayed.

Sample	Molecular Sex	Haplogroup	Is the Haplogroup Found in the AADR Database Mallick et al., 2024) [53]?	Is the Haplogroup Found in the Present-Day Jewish Population [60]?
**ROQ1** **ROQ5**	Male Female	L2a1c + 16129	No, but L2a1I2a was found in Erfurt Jews [20].	Not mentioned.
**ROQ2**	Male	U4a2	Yes. See the ancient distribution of U4a2 in Supplementary Material (Table S7).	Not mentioned.
**ROQ3**	Female	J1c1f	No, but J1c1 distribution is available in Cuesta-Aguirre et al. (2025) [61].	J1c1 in Ashkenazi Jews from Belarus and Russia, and other European countries, including Italy and France.
**ROQ4**	Male	M1a1b1c	No, but M1a1b1 in Spain and Sardinia Bronze Age individuals (see ancient distribution in Cuesta-Aguirre et al., 2025) [61].	M1a1b1c in Ashkenazi Jews from Germany and Poland.
**ROQ7**	Male	R0a4	No. See the ancient distribution of R0a in Supplementary Material (Table S8).	R0a4 in Ashkenazi Jews from the Netherlands, Germany, France, Hungary, Romania, Poland, Lithuania, Belarus, Ukraine, and Russia.
**ROQ10** **ROQ13**	Female Female	H1bo	No.	H1bo in Jewish people from Germany, Poland, Ukraine, Belarus, Greece, and Romania, and in Sephardic Jews.
**ROQ11**	Female	H20a1a	No, but H20a has been detected in Israel (2340–2146 BCE) [62], Turkey (1954–1722 BCE) [63], and Spain (Islamic, Granada, 1000–1100 CE) [64].	H20 in Armenia and the Jewish population from Turkey. H20a1 in Iran and Lebanon. H20a1 in Turkey and the Arab Emirates.
**ROQ12**	Female	K1a1b1a	Yes. It has been found in 11 unrelated Erfurt Jews [20].	The most common haplogroup in the Ashkenazi Jewish population. Present in the Cochin Jewish population (India). Present in Jewish individuals or individuals of Jewish ancestry among Sephardic Jews from Bulgaria, North Macedonia, and Turkey. Observed among descendants of Sephardic conversos
**ROQ15**	Female	U5a2b	Yes. See the ancient distribution of U5a2b in Supplementary Material (Table S9). U5a2b was found in one Erfurt Jew [20].	U5a2b2a in Ashkenazi Jews from Romania, Ukraine, and Belarus

**Table 3:** Y-chromosome haplogroups detected in the males of Roquetes. Their ancient distribution is based on the Allen Ancient DNA Resource (AADR) [53] v62.0.

Sample	Haplogroup (ISOGG 2020)	Haplogroup (SNP-Based)	Is the Haplogroup Found in the AADR Database [53]?
**ROQ1**	J2a2a1~*	J-Y31564	See the ancient distribution of J2a2a in Supplementary Material (Table S11).
**ROQ2**	E1b1b1a1a1c1b1a1a~	E-CTS9507	See the ancient distribution of E1b1b in Supplementary Material (Table S12). E1b1b was found in Norwich (N = 1) [21] and Erfurt (N = 2) [20] Jews.
**ROQ4**	G1a1a1b2b	G-PH1944 (G-FT19393)	G1a1a lineages appeared in Chalcolithic and Bronze Age Iran [65,66] and 15–17th-century Turkey [66].
**ROQ7**	E2a~*	E-Y231455	The E2a haplogroup is found in an ancient individual (891–987 CE) from Kenya [67] and in a present-day individual from the Dinka (South Sudan) [68]. E2a1 is found in two present-day individuals, one from Dinka (South Sudan) [69] and the other from Masai (Kenya) [69].

* Lineage obtained from the ISOGG 2020 tree for the penultimate lineage obtained with Yleaf (See Supplementary Material, Figures S5 and S8), since the last one did not appear on the ISOGG 2020 database.

## Data Availability

FASTQ files for each library are available through the European Nucleotide Archive (ENA), BioProject: PRJEB107193.

## Supplementary Materials

The following supporting information can be downloaded at: https://www.mdpi.com/article/10.3390/genes17030358/s1, Figure S1: Individual ROQ2 karyotype with the homozygous regions highlighted in red. Figure S2: Individual ROQ3 karyotype with the homozygous regions highlighted in red. Figure S3: Individual ROQ4 karyotype with the homozygous regions highlighted in red. Figure S4: Individual ROQ13 karyotype with the homozygous regions highlighted in red. Figure S5: Phylogenetic tree of the Y-chromosome haplogroup corresponding to individual ROQ1. Figure S6: Phylogenetic tree of the Y-chromosome haplogroup corresponding to individual ROQ2. Figure S7: Phylogenetic tree of the Y-chromosome haplogroup corresponding to individual ROQ4. Figure S8: Phylogenetic tree of the Y-chromosome haplogroup corresponding to individual ROQ7. Figure S9: Multidimensional scaling (MDS) based on the 1—outgroup *f*_3_ in the form *f*_3_(Pop1, Pop2; Mbuti) for the populations employed in the present study including the outlier populations (Cochin Jews and ancient Norwich (UK) Jews). The labels EU and ME refer to medieval Jewish individuals from Erfurt from European and Middle Eastern descent, respectively; and the label Israel Middle/Late Bronze Age refers to individuals from Canaan (territory of present-day Israel in the MLBA). Table S1. Information for each sample, including the corresponding archaeological code and grave; whether they were valid for shotgun and the corresponding 5′ C to T first base changes, the sex determination rates and the total of SNPs recovered from the 1240k panel; whether they were valid for human DNA capture and the corresponding 5′ C to T first base changes, the ContaMix assesment and the total of SNPs recovered from the 1240k panel; whether they were employed for genomic analyses; the genetic sex determination; and the uniparental markers’ haplogroups. Table S2. Labels and relevant information for the individuals used in the population analyses. Individuals not included in a given analysis are indicated as “not used” in the corresponding column. Table S3. Overview of sequencing and mapping statistics for the shotgun. The table includes details on sample identifiers, bone types, sequencing metrics (e.g., total reads, mapped reads, endogenous DNA percentage), DNA damage patterns, and related metrics. Libraries with a percentage of damage in the first base lower than 5% are indicated in red and were discarded. Table S4. Overview of sequencing and mapping statistics for the capture. The table includes details on sample identifiers, bone types, sequencing metrics (e.g., total reads, mapped reads, endogenous DNA percentage), DNA damage patterns, and related metrics. Table S5. Pairwise genetic relationship analysis of samples from Roquetes for samples with more than 10,000 autosomal SNPs from the 1240k panel. The analysis is based on SNP mismatch rates calculated using the BREADR algorithm [52]. Table S6. Mitochondrial DNA information for each sample, including the haplogroup, haplotype, and the range of bases with coverage higher than 3X. Additionally, the quality of the haplogroup determination and the percentage of recovered bases (out of the 16,569 bp of the mitochondrial genome) are indicated. Positions in the haplotype are highlighted based on Haplogrep 3 annotations: purple for hotspots, orange for local private mutations, and blue for global private mutations. Table S7. All samples belonging to mtDNA haplogroup U4a2, from the present study, and found in the Ancient Allen database. Roquetes samples are highlighted in maroon, and samples from the Iberian Peninsula are highlighted in bold. Table S8. All samples belonging to mtDNA haplogroup R0a (and their subhaplogroups) from the present study and found in the Ancient Allen database. The sample from Roquetes is highlighted in maroon, and samples from the Iberian Peninsula are highlighted in bold. Table S9. All samples belonging to mtDNA haplogroup U5a2b, from the present study, and found in the Ancient Allen database. The sample from Roquetes is highlighted in maroon, and samples from the Iberian Peninsula are highlighted in bold. Table S10. Y-chromosome haplogroup assignments for male samples based on Yleaf v3.2.1 [51]. Table S11. All samples belonging to Y-chromosome haplogroup J2a2a (and their subhaplogroups) from the present study and found in the Ancient Allen database. The sample from Roquetes is highlighted in maroon, and samples from the Iberian Peninsula are highlighted in bold. Table S12. All samples belonging to Y-chromosome haplogroup E1b1b (and their subhaplogroups) from the present study and found in the Ancient Allen database. The sample from Roquetes is highlighted in maroon, and samples from the Iberian Peninsula are highlighted in bold. Table S13. Results of the outgroup *f*_3_-statistics analysis for the Roquetes population (ROQ). The table includes the *f*_3_ values, standard errors, Z-scores, and the number of SNPs used in each comparison. The labels Germany_Jewish_13-15_EU and Germany_Jewish_13-15_ME refer to medieval Jewish individuals from Erfurt from European and Middle Eastern descent, respectively; and the label Israel Middle/Late Bronze Age refers to individuals from Canaan (territory of present-day Israel in the MLBA). Table S14. Results of the outgroup *f*_3_-statistics analysis for the pair of populations. The table includes the *f*_3_ values, standard errors, Z-scores, and the number of SNPs used in each comparison. The labels Germany_Jewish_13-15_EU and Germany_Jewish_13-15_ME refer to medieval Jewish individuals from Erfurt from European and Middle Eastern descent, respectively; and the label Israel Middle/Late Bronze Age refers to individuals from Canaan (territory of present-day Israel in the MLBA). These results were employed to generate the multidimensional scaling plot (Figure 6 and Supplementary Material, Figure S9). Table S15. All qpAdm models tested for the Roquetes population (ROQ) analyzed in the present study. Models with a *p*-value below 0.05 and two-way models with an ancestry proportion (C) below 0.1 were discarded. Models with negative values after subtracting two standard deviations from the ancestry proportion (C - 2sd < 0) were also discarded. Values that fail those filters are highlighted in red, and the whole discarded model is marked in pink. The labels Germany_Jewish_13-15_EU and Germany_Jewish_13-15_ME refer to medieval Jewish individuals from Erfurt from European and Middle Eastern descent, respectively; and the label Israel Middle/Late Bronze Age refers to individuals from Canaan (territory of present-day Israel in the MLBA).

## References

[1] Goodman M. (2018). A History of Judaism.

[2] Lederhendler Grabbe L.L. (2004). A History of the Jews and Judaism in the Second Temple Period.

[3] Na’aman N. (1994). The Canaanites and Their Land, Ugarit-Forschungen 26; Internationales Jahrbuch für die Alertumskunde Syrien-Palästinas.

[4] Ben-Sasson H.H., Ben-Sasson H.H. (1976). A History of the Jewish People.

[5] Na’aman N. (2005). Ancient Israel and Its Neighbors Interaction and Counteraction: Collected Essays.

[6] Frishman A. (2008). The Early Ashkenazi Jews: Since Their Settlement in North-West Europe to the First Crusade.

[7] Benbassa E., Rodrigue A. (2000). Sephardi Jewry: A History of the Judeo-Spanish Community, 14th–20th Centuries.

[8] Baer Y. (1961). A History of the Jews in Christian Spain.

[9] Valdeón J. (2004). Cristianos, Musulmanes y Judíos en la España Medieval: De la Aceptación al Rechazo.

[10] Netanyahu B. (1995). The Origins of the Inquisition in Fifteenth Century Spain.

[11] Nirenberg D. (1996). Communities of Violence.

[12] Greber J.S. (1992). The Jews of Spain: A History of the Sephardic Experience. AJS Rev..

[13] Assis Y.T. (1997). The Golden Age of Aragonese Jewry: Community and Society in the Crown of Aragon, 1213–1327.

[14] Feliu i Mabres E. (2010). Lletres Hebrees a la Barcelona Medieval.

[15] Rumeu de Armas A. (1985). Nueva luz Sobre las Capitulaciones de Santa Fe de 1492 Concertadas Entre los Reyes Católicos y Cristóbal Colón: Estudio Institucional y Diplomático.

[16] Colet A., Muntané i Sant J.X., Green M.H. (2015). The Black Death and Its Consequences for the Jewish Community in Tàrrega: Lessons from History and Archeology. Pandemic Disease in the Medieval World: Rethinking the Black Death.

[17] Colet A., Muntané J.X., Saula O., Ruiz J., Subira De Galdacano M.E. (2010). La necròpolis medieval jueva de les Roquetes (Tàrrega, Urgell). Tribuna d’Arqueologia, 2008–2009.

[18] Ruiz J., Subirà M.E., Colet A., Muntané J.X., Saula O., Ruiz J., Subira De Galdacano M.E. (2010). L’antropologia a la necròpolis medieval jueva de les Roquetes. Tribuna d’Arqueologia, 2008–2009.

[19] Ostrer H., Skorecki K. (2013). The population genetics of the Jewish people. Hum. Genet..

[20] Waldman S., Backenroth D., Harney É., Flohr S., Neff N.C., Buckley G.M., Fridman H., Akbari A., Rohland N., Mallick S. (2022). Genome-wide data from medieval German Jews show that the Ashkenazi founder event pre-dated the 14th century. Cell.

[21] Brace S., Diekmann Y., Booth T., Macleod R., Timpson A., Stephen W., Emery G., Cabot S., Thomas M.G., Barnes I. (2022). Genomes from a medieval mass burial show Ashkenazi-associated hereditary diseases pre-date the 12th century. Curr. Biol..

[22] Atzmon G., Hao L., Pe’er I., Velez C., Pearlman A., Palamara P.F., Morrow B., Friedman E., Oddoux C., Burns E. (2010). Abraham’s children in the genome era: Major Jewish diaspora populations comprise distinct genetic clusters with shared Middle Eastern Ancestry. Am. J. Hum. Genet..

[23] Vinueza-Espinosa D.C., Santos C., Martínez-Labarga C., Malgosa A. (2020). Human DNA extraction from highly degraded skeletal remains: How to find a suitable method?. Electrophoresis.

[24] Kapp J.D., Green R.E., Shapiro B. (2021). A Fast and Efficient Single-stranded Genomic Library Preparation Method Optimized for Ancient DNA. J. Hered..

[25] Kircher M., Sawyer S., Meyer M. (2012). Double indexing overcomes inaccuracies in multiplex sequencing on the Illumina platform. Nucleic Acids Res..

[26] Mathieson I., Lazaridis I., Rohland N., Mallick S., Patterson N., Roodenberg S.A., Harney E., Stewardson K., Fernandes D., Novak M. (2015). Genome-wide patterns of selection in 230 ancient Eurasians. Nature.

[27] Fellows Yates J.A., Lamnidis T.C., Borry M., Andrades Valtueña A., Fagernäs Z., Clayton S., Garcia M.U., Neukamm J., Peltzer A. (2021). Reproducible, portable, and efficient ancient genome reconstruction with nf-core/eager. PeerJ.

[28] Ewels P.A., Peltzer A., Fillinger S., Patel H., Alneberg J., Wilm A., Garcia M.U., Di Tommaso P., Nahnsen S. (2020). The nf-core framework for community-curated bioinformatics pipelines. Nat. Biotechnol..

[29] Schubert M., Lindgreen S., Orlando L. (2016). AdapterRemoval v2: Rapid adapter trimming, identification, and read merging. BMC Res. Notes.

[30] Li H., Durbin R. (2009). Fast and accurate short read alignment with Burrows–Wheeler transform. Bioinformatics.

[31] Oliva A., Tobler R., Cooper A., Llamas B., Souilmi Y. (2021). Systematic benchmark of ancient DNA read mapping. Brief. Bioinform..

[32] Oliva A., Tobler R., Llamas B., Souilmi Y. (2021). Additional evaluations show that specific BWA-aln settings still outperform BWA-mem for ancient DNA data alignment. Ecol. Evol..

[33] Danecek P., Bonfield J.K., Liddle J., Marshall J., Ohan V., Pollard M.O., Whitwham A., Keane T., McCarthy S.A., Davies R.M. (2021). Twelve years of SAMtools and BCFtools. GigaScience.

[34] (2019). Picard Toolkit. Broad Institute. https://broadinstitute.github.io/picard/.

[35] Skoglund P., Northoff B.H., Shunkov M.V., Derevianko A.P., Pääbo S., Krause J., Jakobsson M. (2014). Separating endogenous ancient DNA from modern day contamination in a Siberian Neandertal. Proc. Natl. Acad. Sci. USA.

[36] Jun G., Wing M.K., Abecasis G.R., Kang H.M. (2015). An efficient and scalable analysis framework for variant extraction and refinement from population-scale DNA sequence data. Genome Res..

[37] Okonechnikov K., Conesa A., García-Alcalde F. (2016). Qualimap 2: Advanced multi-sample quality control for high-throughput sequencing data. Bioinformatics.

[38] Lamnidis T.C., Majander K., Jeong C., Salmela E., Wessman A., Moiseyev V., Khartanovich V., Balanovsky O., Ongyerth M., Weihmann A. (2018). Ancient Fennoscandian genomes reveal origin and spread of Siberian ancestry in Europe. Nat. Commun..

[39] Fowler C., Olalde I., Cummings V., Armit I., Büster L., Cuthbert S., Rohland N., Cheronet O., Pinhasi R., Reich D. (2022). A high-resolution picture of kinship practices in an Early Neolithic tomb. Nature.

[40] Fu Q., Mittnik A., Johnson P.L.F., Bos K., Lari M., Bollongino R., Sun C., Giemsch L., Schmitz R., Burger J. (2013). A Revised Timescale for Human Evolution Based on Ancient Mitochondrial Genomes. Curr. Biol..

[41] Andrews R.M., Kubacka I., Chinnery P.F., Lightowlers R.N., Turnbull D.M., Howell N. (1999). Reanalysis and revision of the Cambridge reference sequence for human mitochondrial DNA. Nat. Genet..

[42] Peltzer A., Jäger G., Herbig A., Seitz A., Kniep C., Krause J., Nieselt K. (2016). EAGER: Efficient ancient genome reconstruction. Genome Biol..

[43] Garrison E., Marth G. (2012). Haplotype-based variant detection from short-read sequencing. arXiv.

[44] Garrison E., Kronenberg Z.N., Dawson E.T., Pedersen B.S., Prins P. (2022). A spectrum of free software tools for processing the VCF variant call format: Vcflib, bio-vcf, cyvcf2, hts-nim and slivar. PLoS Comput. Biol..

[45] Schönherr S., Weissensteiner H., Kronenberg F., Forer L. (2023). Haplogrep 3—An interactive haplogroup classification and analysis platform. Nucleic Acids Res..

[46] Robinson J.T., Thorvaldsdóttir H., Winckler W., Guttman M., Lander E.S., Getz G., Mesirov J.P. (2011). Integrative genomics viewer. Nat. Biotechnol..

[47] Soares P., Ermini L., Thomson N., Mormina M., Rito T., Röhl A., Salas A., Oppenheimer S., Macaulay V., Richards M.B. (2009). Correcting for Purifying Selection: An Improved Human Mitochondrial Molecular Clock. Am. J. Hum. Genet..

[48] Li M., Schröder R., Ni S., Madea B., Stoneking M. (2015). Extensive tissue-related and allele-related mtDNA heteroplasmy suggests positive selection for somatic mutations. Proc. Natl. Acad. Sci. USA.

[49] Cuesta-Aguirre D.R., Amor-Jimenez C., Malgosa A., Santos C. (2025). A Post-Mortem Molecular Damage Profile in the Ancient Human Mitochondrial DNA. Mol. Ecol. Resour..

[50] Cuesta-Aguirre D.R., Onieva A., Aluja M.P., Santos C. (2026). Probability of Mitochondrial DNA heteroplasmy in different tissues from European populations. Mitochondrion.

[51] Ralf A., Montiel González D., Zhong K., Kayser M. (2018). Yleaf: Software for Human Y-Chromosomal Haplogroup Inference from Next-Generation Sequencing Data. Mol. Biol. Evol..

[52] Rohrlach A.B., Tuke J., Popli D., Haak W. (2023). BREADR: An R Package for the Bayesian Estimation of Genetic Relatedness from Low-coverage Genotype Data. BioRxiv.

[53] Mallick S., Micco A., Mah M., Ringbauer H., Lazaridis I., Olalde I., Patterson N., Reich D. (2024). The Allen Ancient DNA Resource (AADR) a curated compendium of ancient human genomes. Sci. Data.

[54] Alexander D.H., Novembre J., Lange K. (2009). Fast model-based estimation of ancestry in unrelated individuals. Genome Res..

[55] R Core Team (2024). R: A Language and Environment for Statistical Computing.

[56] Wickham H. (2016). ggplot2: Elegant Graphics for Data Analysis.

[57] Behr A.A., Liu K.Z., Liu-Fang G., Nakka P., Ramachandran S. (2016). pong: Fast analysis and visualization of latent clusters in population genetic data. Bioinformatics.

[58] Harney É., Patterson N., Reich D., Wakeley J. (2021). Assessing the performance of qpAdm: A statistical tool for studying population admixture. Genetics.

[59] Ringbauer H., Novembre J., Steinrücken M. (2021). Parental relatedness through time revealed by runs of homozygosity in ancient DNA. Nat. Commun..

[60] Brook K.A. (2022). The Maternal Genetic Lineages of Ashkenazic Jews.

[61] Cuesta-Aguirre D.R., Campoy-Caballero M.R., Sandoval-Ávila C., Busquets i Costa C., Fàbregas i Espadaler M., Sinner A.G., de Prado G., Molist Capella N., Duran i Caixal M., Mestres Santacreu I. (2025). Mitochondrial DNA diversity in northeast Iberians during the Iron Age. J. Archaeol. Sci..

[62] Agranat-Tamir L., Waldman S., Martin M.A.S., Gokhman D., Mishol N., Eshel T., Cheronet O., Rohland N., Mallick S., Adamski N. (2020). The Genomic History of the Bronze Age Southern Levant. Cell.

[63] Skourtanioti E., Erdal Y.S., Frangipane M., Balossi Restelli F., Yener K.A., Pinnock F., Matthiae P., Özbal R., Schoop U.-D., Guliyev F. (2020). Genomic History of Neolithic to Bronze Age Anatolia, Northern Levant, and Southern Caucasus. Cell.

[64] Olalde I., Mallick S., Patterson N., Rohland N., Villalba-Mouco V., Silva M., Dulias K., Edwards C.J., Gandini F., Pala M. (2019). The genomic history of the Iberian Peninsula over the past 8000 years. Science.

[65] Lazaridis I., Nadel D., Rollefson G., Merrett D.C., Rohland N., Mallick S., Fernandes D., Novak M., Gamarra B., Sirak K. (2016). Genomic insights into the origin of farming in the ancient Near East. Nature.

[66] Lazaridis I., Alpaslan-Roodenberg S., Acar A., Açıkkol A., Agelarakis A., Aghikyan L., Akyüz U., Andreeva D., Andrijašević G., Antonović D. (2022). Ancient DNA from Mesopotamia suggests distinct Pre-Pottery and Pottery Neolithic migrations into Anatolia. Science.

[67] Prendergast M.E., Lipson M., Sawchuk E.A., Olalde I., Ogola C.A., Rohland N., Sirak K.A., Adamski N., Bernardos R., Broomandkhoshbacht N. (2019). Ancient DNA reveals a multistep spread of the first herders into sub-Saharan Africa. Science.

[68] Meyer M., Kircher M., Gansauge M.-T., Li H., Racimo F., Mallick S., Schraiber J.G., Jay F., Prüfer K., de Filippo C. (2012). A High-Coverage Genome Sequence from an Archaic Denisovan Individual. Science.

[69] Mallick S., Li H., Lipson M., Mathieson I., Gymrek M., Racimo F., Zhao M., Chennagiri N., Nordenfelt S., Tandon A. (2016). The Simons Genome Diversity Project: 300 genomes from 142 diverse populations. Nature.

[70] (1971). Encyclopaedia Judaica.

[71] Picornell A., Giménez P., Castro J.A., Ramon M.M. (2006). Mitochondrial DNA sequence variation in Jewish populations. Int. J. Leg. Med..

[72] Nogueiro I., Teixeira J.C., Amorim A., Gusmão L., Alvarez L. (2015). Portuguese crypto-Jews: The genetic heritage of a complex history. Front. Genet..

[73] Thomas M.G., Weale M.E., Jones A.L., Richards M., Smith A., Redhead N., Torroni A., Scozzari R., Gratrix F., Tarekegn A. (2002). Founding Mothers of Jewish Communities: Geographically Separated Jewish Groups Were Independently Founded by Very Few Female Ancestors. Am. J. Hum. Genet..

[74] Behar D.M., Metspalu E., Kivisild T., Rosset S., Tzur S., Hadid Y., Yudkovsky G., Rosengarten D., Pereira L., Amorim A. (2008). Counting the Founders: The Matrilineal Genetic Ancestry of the Jewish Diaspora. PLoS ONE.

[75] Ferragut J.F., Ramon C., Castro J.A., Amorim A., Alvarez L., Picornell A. (2020). Middle eastern genetic legacy in the paternal and maternal gene pools of Chuetas. Sci. Rep..

[76] Behar D.M., Metspalu E., Kivisild T., Achilli A., Hadid Y., Tzur S., Pereira L., Amorim A., Quintana-Murci L., Majamaa K. (2006). The Matrilineal Ancestry of Ashkenazi Jewry: Portrait of a Recent Founder Event. Am. J. Hum. Genet..

[77] Narasimhan V.M., Patterson N., Moorjani P., Rohland N., Bernardos R., Mallick S., Lazaridis I., Nakatsuka N., Olalde I., Lipson M. (2019). The formation of human populations in South and Central Asia. Science.

[78] Behar D.M., Garrigan D., Kaplan M.E., Mobasher Z., Rosengarten D., Karafet T.M., Quintana-Murci L., Ostrer H., Skorecki K., Hammer M.F. (2004). Contrasting patterns of Y chromosome variation in Ashkenazi Jewish and host non-Jewish European populations. Hum. Genet..

[79] Adams S.M., Bosch E., Balaresque P.L., Ballereau S.J., Lee A.C., Arroyo E., López-Parra A.M., Aler M., Grifo M.S.G., Brion M. (2008). The Genetic Legacy of Religious Diversity and Intolerance: Paternal Lineages of Christians, Jews, and Muslims in the Iberian Peninsula. Am. J. Hum. Genet..

[80] Nogueiro I., Manco L., Gomes V., Amorim A., Gusmão L. (2010). Phylogeographic analysis of paternal lineages in NE Portuguese Jewish communities. Am. J. Phys. Anthropol..

[81] Assis Y.T., Meyerson M., Chazan R. (2018). The Iberian Peninsula. The Cambridge History of Judaism.

[82] García-Arenal M., Wiegers G.A., Szpiech R. (2019). Interreligious Encounters in Polemics Between Christians, Jews, and Muslims in Iberia and Beyond.

[83] Nirenberg D. (2002). Mass Conversion and Genealogical Mentalities: Jews and Christians in Fifteenth-Century Spain. Past Present.

[84] Listman J.B., Hasin D., Kranzler H.R., Malison R.T., Mutirangura A., Sughondhabirom A., Aharonovich E., Spivak B., Gelernter J. (2010). Identification of population substructure among Jews using STR markers and dependence on reference populations included. BMC Genet..

[85] Bray S.M., Mulle J.G., Dodd A.F., Pulver A.E., Wooding S., Warren S.T. (2010). Signatures of founder effects, admixture, and selection in the Ashkenazi Jewish population. Proc. Natl. Acad. Sci. USA.

[86] Kopelman N.M., Stone L., Wang C., Gefel D., Feldman M.W., Hillel J., Rosenberg N.A. (2009). Genomic microsatellites identify shared Jewish ancestry intermediate between Middle Eastern and European populations. BMC Genet..

[87] Campbell C.L., Palamara P.F., Dubrovsky M., Botigué L.R., Fellous M., Atzmon G., Oddoux C., Pearlman A., Hao L., Henn B.M. (2012). North African Jewish and non-Jewish populations form distinctive, orthogonal clusters. Proc. Natl. Acad. Sci. USA.

[88] Behar D.M., Yunusbayev B., Metspalu M., Metspalu E., Rosset S., Parik J., Rootsi S., Chaubey G., Kutuev I., Yudkovsky G. (2010). The genome-wide structure of the Jewish people. Nature.

[89] Álvarez-Álvarez M.M., Risch N., Gignoux C.R., Huntsman S., Ziv E., Fejerman L., Esteban M.E., Gayà-Vidal M., Sobrino B., Brisighelli F. (2018). Genetic analysis of Sephardic ancestry in the Iberian Peninsula. bioRxiv.

