# Supplementary material for: Uncovering a Medieval Pogrom: Genetic History of a Jewish Community in Catalonia (Spain)
Source: Genes (Basel). 2026 Mar 23;17(3):358. doi: 10.3390/genes17030358 (PMC13026783; doi:10.3390/genes17030358)
Supplement: Supplementary file 1 [file genes-17-00358-s001.zip › Supplementary_material_figures.pdf]

Supplementary figures

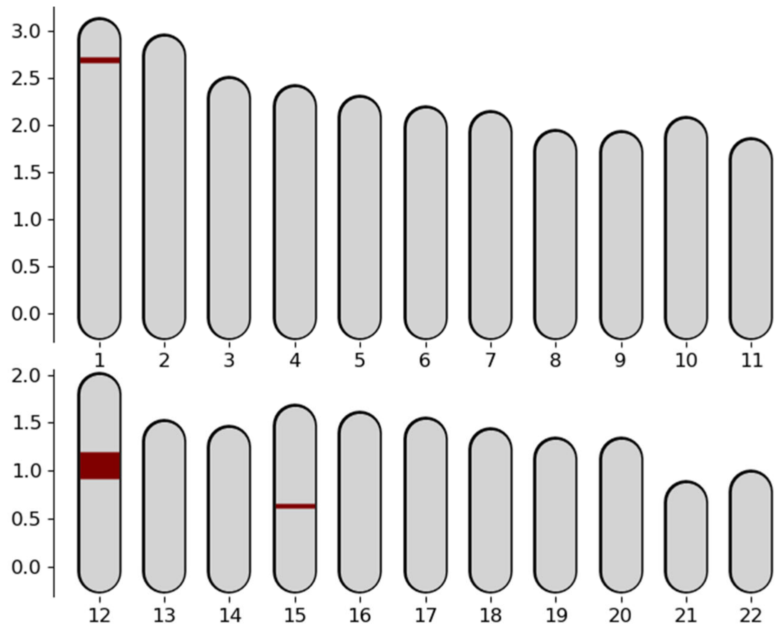

Figure S1: Individual ROQ2 karyotype with the homozygous regions highlighted in red.

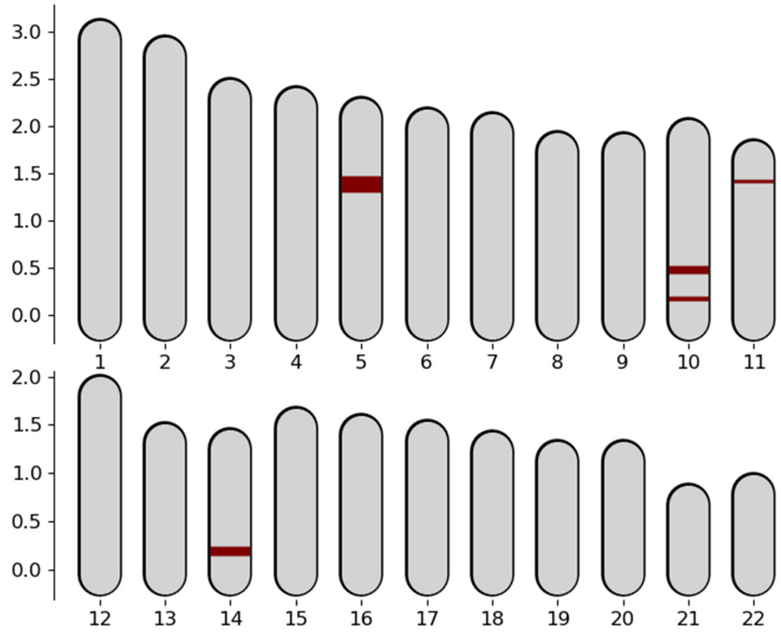

Figure S2: Individual ROQ3 karyotype with the homozygous regions highlighted in red.

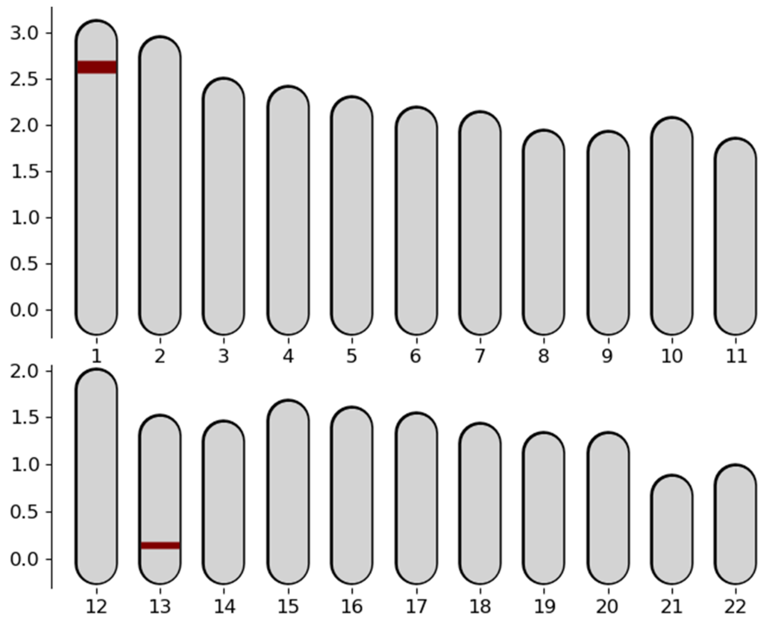

Figure S3: Individual ROQ4 karyotype with the homozygous regions highlighted in red.

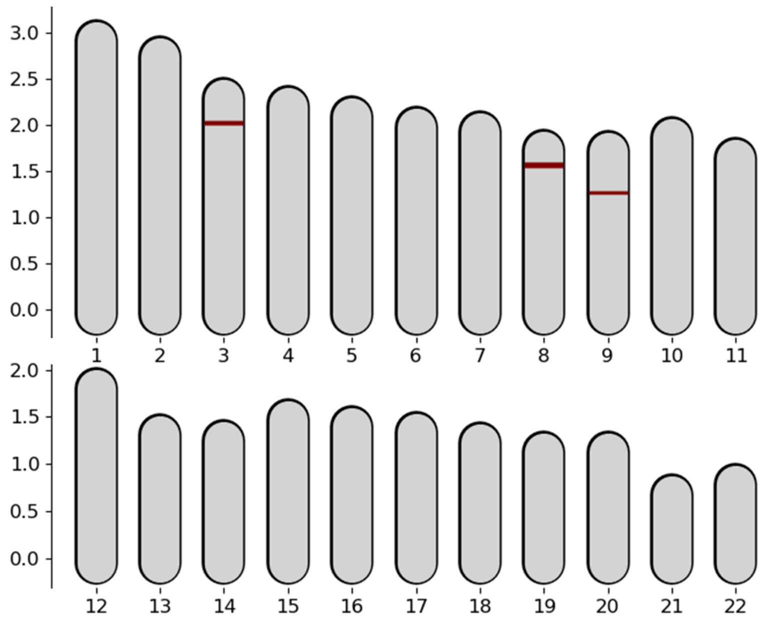

Figure S4: Individual ROQ13 karyotype with the homozygous regions highlighted in red.

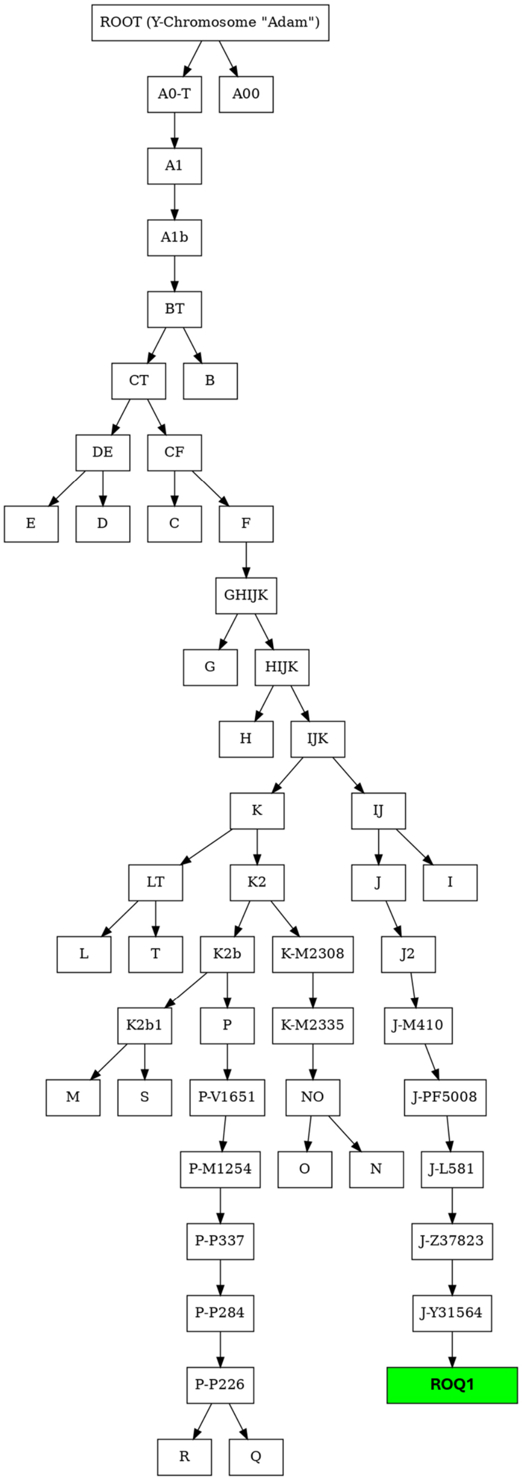

Figure S5: Phylogenetic tree of the Y-chromosome haplogroup corresponding to individual ROQ1.

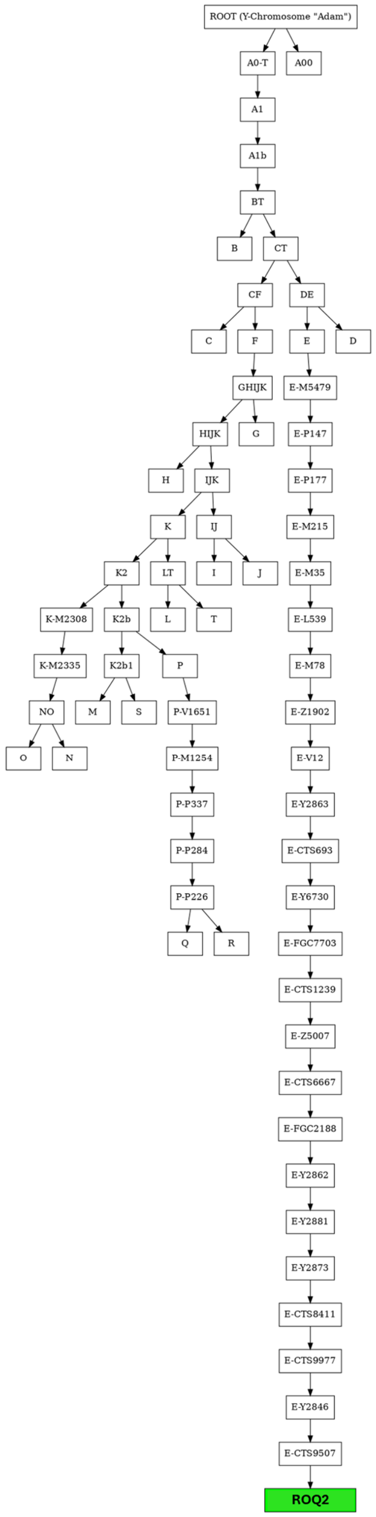

Figure S6: Phylogenetic tree of the Y-chromosome haplogroup corresponding to individual ROQ2.

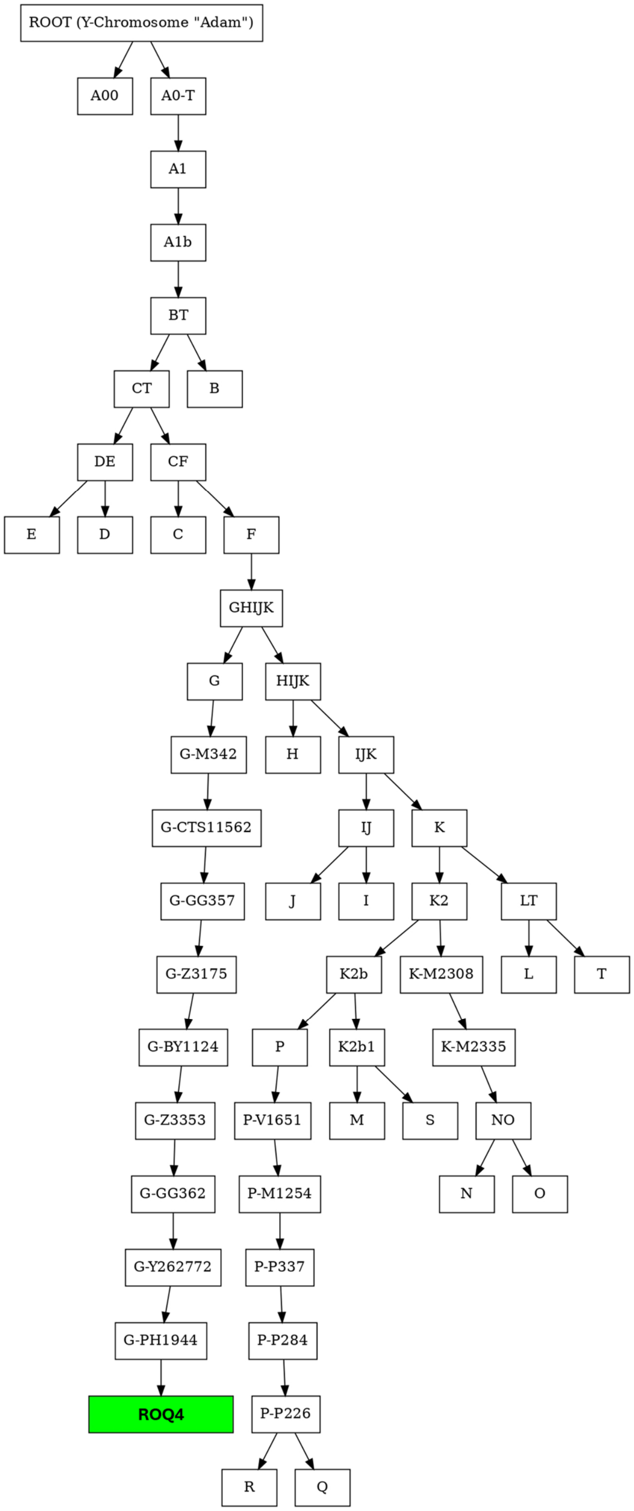

Figure S7: Phylogenetic tree of the Y-chromosome haplogroup corresponding to individual ROQ4.

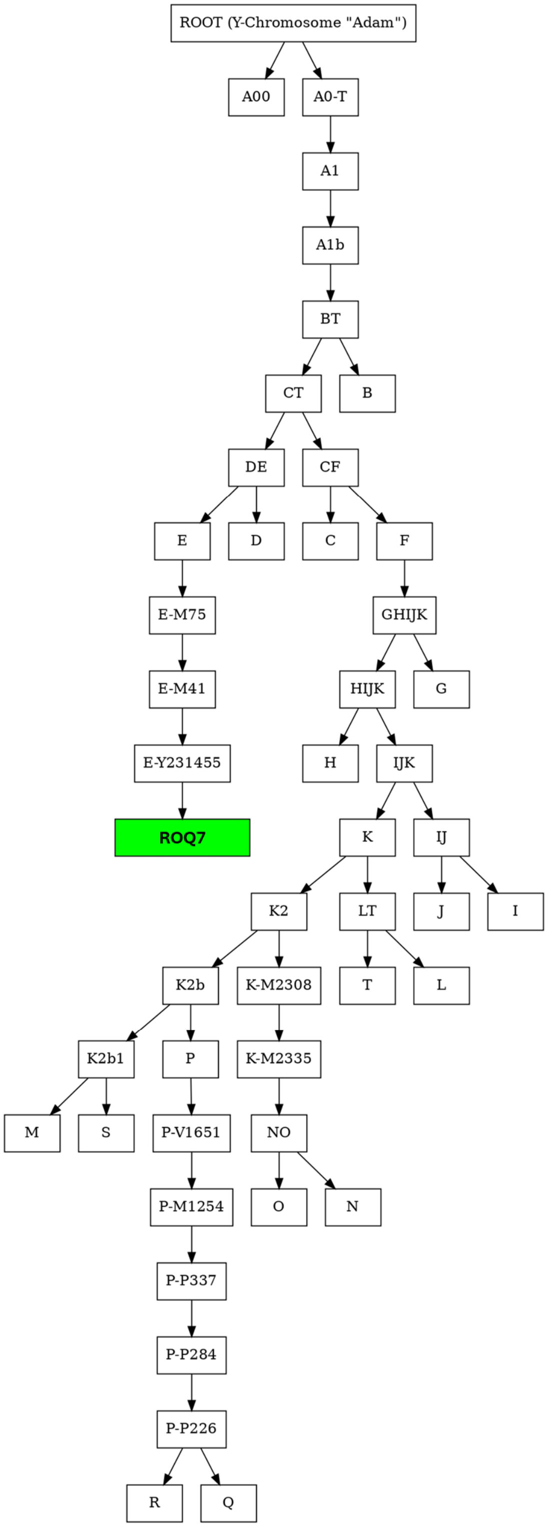

Figure S8: Phylogenetic tree of the Y-chromosome haplogroup corresponding to individual ROQ7.

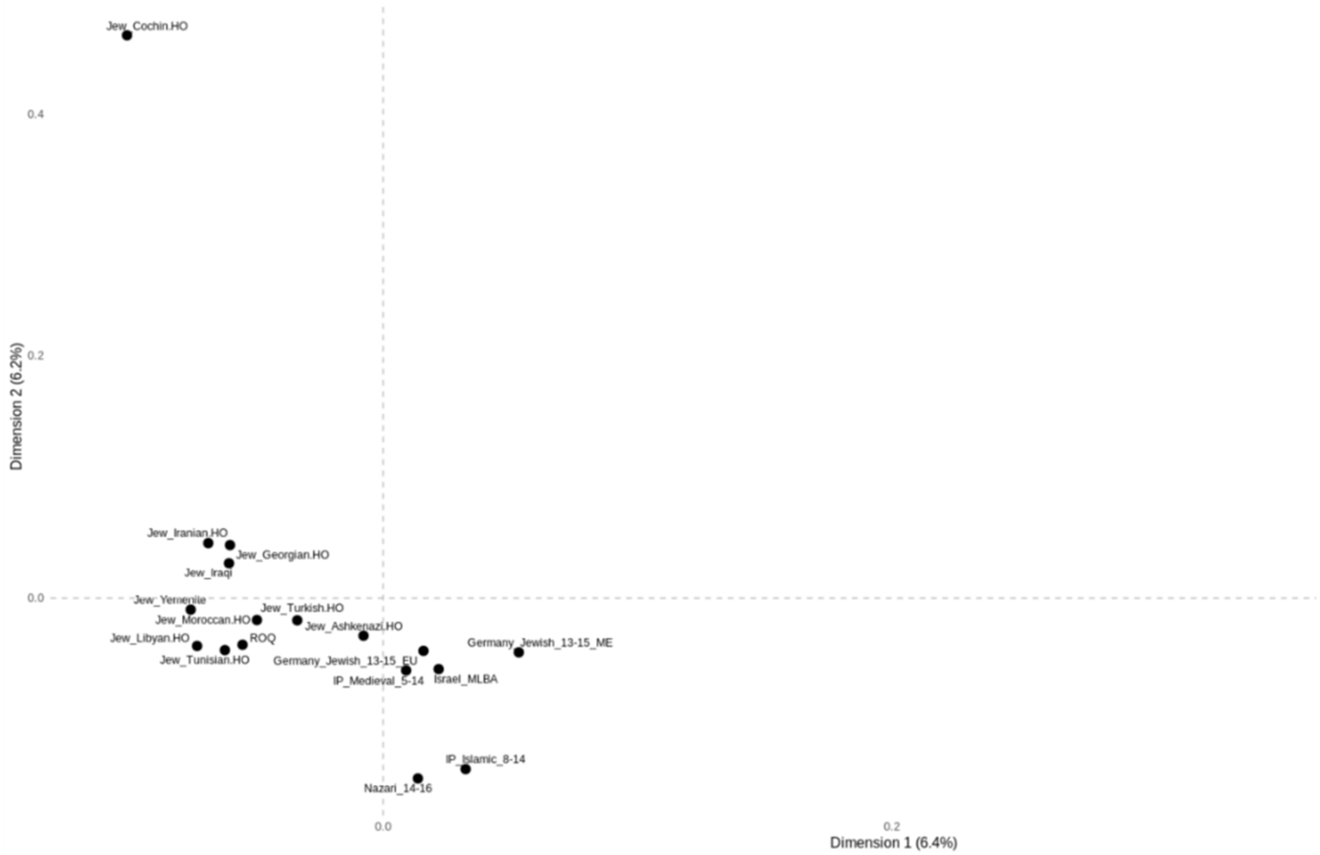

Figure S9: Multidimensional scaling (MDS) based on the 1 – outgroup f_3 in the form f_3(Pop1, Pop2; Mbuti) for the populations employed in the present study including the outlier populations (Cochin Jews and ancient Norwich (UK) Jews). The labels EU and ME refer to medieval Jewish individuals from Erfurt from European and Middle Eastern descent, respectively; and the label Israel Middle/Late Bronze Age refers to individuals from Canaan (territory of present-day Israel in the MLBA).
